# Young Canadian e-Cigarette Users and the COVID-19 Pandemic: Examining Vaping Behaviors by Pandemic Onset and Gender

**DOI:** 10.3389/fpubh.2020.620748

**Published:** 2021-01-27

**Authors:** D. Brett Hopkins, Mohammed Al-Hamdani

**Affiliations:** ^1^Department of Community Health and Epidemiology, Dalhousie University, Halifax, NS, Canada; ^2^The Lung Association of Nova Scotia, Halifax, NS, Canada; ^3^Department of Psychology, Saint Mary's University, Halifax, NS, Canada

**Keywords:** electronic cigarette, coronavirus, teenager, vaper, substance use

## Abstract

The aim of this study was to test how youth and young adult e-cigarette users responded to the COVID-19 pandemic. The 2020 *Youth and Young Adult Vaping Survey* (*N* = 1,308) included 540 (44.7%) participants that reported differences in their vaping behaviors since the onset of the pandemic. Gender was the only relevant covariate that yielded a significant effect and/or interaction through a multivariate test. A two-way multivariate analysis of variance was used to test the effect of pandemic onset (pre- vs. during-pandemic), gender (males vs. females), and their interaction on vaping behaviors (days of vaping per week, episodes of vaping per day, and puffs per vaping episode). Respondents reported fewer days of vaping per week, episodes of vaping per day, and puffs per vaping episode during-pandemic than pre-pandemic [*F*_(3,533)_ = 52.81, *p* < 0.001, $\eta_{\text{p}}^{2}$ = 0.229]. The multivariate effect of gender on the three vaping outcomes was not statistically significant [*F*_(3, 533)_ = 2.14, *p* = 0.095, $\eta_{\text{p}}^{2}$ = 0.012], though the interaction between pandemic onset and gender was [*F*_(3, 533)_ = 2.86, *p* = 0.036, $\eta_{\text{p}}^{2}$ = 0.016]. Males reported fewer episodes of vaping per day [*t*_(262)_ = 7.40, *p* < 0.001, 95% CI: 5.19–8.97] and puffs per vaping episode [*t*_(263)_ = 3.23, *p* = 0.001, 95% CI:0.292–1.20] during-pandemic than pre-pandemic. Females reported fewer vaping episodes per day during-pandemic than pre-pandemic [*t*_(273)_ = 5.14, *p* < 0.001, 95% CI: 2.76–6.18]. Further, females reported more frequent puffs per vaping episode in comparison to males during-pandemic [*t*_(538)_ = −2.38, *p* = 0.017, 95% CI: −2.09–0.200]. The COVID-19 pandemic presents an opportunity to reduce vaping through health promotion messaging. Since females take more puffs per vaping episode overall, they may benefit the most from greater vaping cessation supports.

## Introduction

The novel SARS-CoV-2 virus and the resulting declaration of the 2020 COVID-19 pandemic sparked discourse concerning vaping and smoking as risk factors for morbidity and mortality of COVID-19 (1–3). These concerns add to a plethora of research from recent years documenting the rise of vaping among youth and young adults (4). Almost without exception, this research is appended with the caution that the findings should be considered in the context that there is a dearth of evidence on the harms of vaping, especially among young and non-smoking persons, and that more research is needed to investigate this issue. The COVID-19 pandemic adds a layer of complexity to this research. Specifically, a population which is at low risk of COVID-19 harms (i.e., youth and young adults) may become more vulnerable by way of vaping behaviors (i.e., hand-to-mouth virus transmission) and its associated respiratory harms (5).

The act of vaping requires repetitive physical contact between a person's hands, mouth, and e-cigarette (6). If an e-cigarette user is exposed to a person or surface with COVID-19, they would presumably be at higher risk of contracting the virus. In some instances, individuals may share their device with others, further increasing the risk of virus transmission (7). In the event that a person is wearing a mask in a public setting, as now recommended in several jurisdictions and by the World Health Organization, they would ultimately have to remove it to use an e-cigarette, which could increase both the risk of exposure and also transmission to others (8, 9). Recent evidence suggests that seeking a COVID-19 test and receiving a positive result was more likely among youth and young adult e-cigarette users compared to non-users, especially among dual cigarette and e-cigarette users (10).

While respiratory harms resulting from smoking have been well-established, such as tuberculosis, lung cancer, COPD, and asthma, evidence on the respiratory harms associated with vaping is scarcer, yet emerging (11). Short-term respiratory symptoms (e.g., cough, phlegm) are more frequently reported among young and adult e-cigarette users (12, 13). Further, many e-cigarette users use flavored products, which contain chemical additives, which may pose yet-to-be-established harms to the lungs (14, 15). Due to the novelty of e-cigarettes, it may require decades of research to establish the long-term biologic harms of vaping.

Emerging evidence suggests that nicotine exposure may exacerbate the pathobiology of COVID-19, namely through its interaction with Angiotensin-converting enzyme 2 (ACE2) (16). More specifically, cigarette and e-cigarette use can stimulate ACE2 receptors in the brain and lungs and put users of these products at higher risk for complications resulting from COVID-19 (17).

### Pandemic Onset and Vaping

The unprecedented COVID-19 pandemic presents a unique opportunity to examine vaping under atypical conditions. Specifically, it allows us to hypothesize how youth and young adults are adapting their vaping behaviors in response to several aspects of daily life interrupted by the pandemic. Youth and young adults are not attending secondary and post-secondary schools and many young adults are working remotely from home. Early evidence suggests that Canadian high school students reduced vaping in the weeks following the recommendation for physical distancing [early April 2020; (18)].

Youth and young adults may use e-cigarettes less during the pandemic in comparison to pre-pandemic for a number of reasons. Vaping behaviors among youth are often hidden from parents and guardians, so youth spending more time at home than at school may limit their opportunity to use e-cigarettes without suspicion (19). Furthermore, youth who are underage and cannot access vaping products traditionally may not be able to meet with older peers or other social sources who purchase products on their behalf (20, 21). There is also the potential that regular users had reduced or no access to e-cigarettes from physical vape stores, which are the primary means of access to e-cigarettes in Canada (22). With respect to physical distancing guidelines, there are also fewer opportunities to meet with peers to socialize, an occasion that facilitates vaping among youth and young adults (23), which is especially true regarding the early months of the pandemic. Finally, vaping may reduce among the whole sample as a result of public health messaging on the risks of vaping during the pandemic (24, 25).

Evidence that concerns how e-cigarette users changed their vaping behaviors after pandemic onset is limited, especially among adolescent populations. One study of youth and young adult (aged 13–24 years) e-cigarette users in the United States reported that more than half (56.4%) of users reported different vaping behaviors since the pandemic onset, with 66.7% of those reporting different behaviors reducing use (26).

### The Current Study

We administered the *2020 Youth and Young Adult Vaping Survey* to Canadian e-cigarette users aged 16–24 during April and May 2020. The first COVID-19 case in Canada was confirmed on February 20, 2020 and the World Health Organization declared a pandemic on March 11, 2020 (27). By the time that our survey was administered, the number of cases in Canada exceeded 20,000 (27). Thus, we had a unique opportunity to ask respondents to report their vaping behaviors prior to learning about the pandemic (retrospective), with the advantage of a limited recall period, and their vaping behaviors after the onset of the pandemic.

The aim of this study is to examine how youth and young adult e-cigarette users responded to the onset of the COVID-19 pandemic with respect to their vaping behaviors. Specifically, our goal is to test the effect of pandemic onset (pre- vs. during-pandemic) on three vaping behaviors: days of vaping per week, episodes of vaping per day, and puffs per vaping episode. The behaviors we chose measure vaping frequency, rather than prevalence. It is well-established that vaping is more prevalent among youth and young adults. However, the vaping behaviors among regular users that we chose are less evidenced and provide more insight into how youth and young adults engage in vaping behaviors rather than a dichotomous confirmation of past-30-days use. All of the aforementioned evidence suggests that youth and young adult e-cigarette users are likely to engage in vaping behaviors less during-pandemic relative to pre-pandemic.

Our study will make notable contributions to the vaping literature by adding evidence that examines multiple vaping behaviors both pre- and during-pandemic time periods. Additionally, we will present our findings in the context of three vaping behaviors, rather than overall use, which will identify which specific aspects of vaping (e.g., number of puffs) changed/did not change. We anticipate that our findings will inform prevention and policy strategies that target regular e-cigarette users with respect to any differences identified through our analysis.

## Methods

### Recruitment

We recruited youth (16–18 years old) and young adult (19–24 years old) e-cigarette users residing in five Canadian provinces (Alberta, British Columbia, Manitoba, Ontario, and Saskatchewan) for the *2020 Youth and Young Adult Vaping Survey*. The aim of this project was to gain insight into the perceptions and experiences of vaping among regular e-cigarette users (at least once/week). A youth and young adult sample was chosen because the prevalence of e-cigarette use in Canada is highest among these age groups compared to persons 25 and older (28).

Recruitment advertisements posted on Facebook and Instagram invited persons interested in the survey to a landing page on Qualtrics, an online survey platform. We enabled Qualtrics' “Prevent Ballot Box Stuffing” feature to limit fraudulent responses (i.e., taking the survey more than once). Participants viewed an online informed consent form and verified eligibility through a series of questions. We verified eligibility by asking if they are regular e-cigarette users (at least once a week over the last 3 months), reside in one of the five listed provinces, and are between the ages of 16 and 24. However, given the online nature of the study and in order to maintain anonymity, we were not able to confirm participants' age or e-cigarette use. An automated process invited eligible participants to complete the survey through Qualtrics. We entered eligible participants in a draw for 1 of 5 $100 gift cards and compensated those that completed the survey in full with $10. Email addresses could only be entered once to discourage participants from trying to take the survey more than once. Ethics approval was obtained from Saint Mary's Research Ethics Board (#19–105).

### Survey

The 2020 Youth and Young Adult Survey was a cross-sectional survey that contained demographic questions and questions about the respondent's vaping behaviors, product preferences, and experiences. Respondents first completed screening questions to verify that they lived in Nova Scotia, were regular e-cigarette users [“Over the past 3 months, have you been vaping regularly (at least once a week)?], and met the age requirements “(What is your age? Please enter the number only).” Respondents selected their gender as “Male,” “Female,” or “Other (please specify).”

For the purpose of this study, respondents reported their vaping behaviors pre-pandemic by responding to three questions: “How many days per week do you vape?,” “On the days you vape, how many times do you use it each day? Please enter a number only (e.g., 5),” and “When taking your vape out of your pocket/purse/backpack, how many puffs do you usually take in a single sitting before putting it away? Please enter a number only (e.g., 5).” Next, respondents reported their during-pandemic vaping behaviors by responding to the question “Since becoming aware of the novel COVID-19 (coronavirus) pandemic, I use my vape” with response options as “Less than before,” “The same as before,” or “More than before.” Respondents who answered with more or less than before were prompted with the same three vaping outcome questions outlined above, but with the preface “Since becoming aware of the novel COVID-19 (coronavirus) pandemic….”

### Data Analysis

For the purpose of this study, we excluded e-cigarette users who used products not containing nicotine. Further, our primary analysis was limited to respondents who indicated different vaping behaviors pre- and during-pandemic. We produced descriptive statistics to report demographic and vaping characteristics among this sample. Gender was found to affect vaping behaviors during a multivariate test, while age category (youth vs. young adult)^1^, employment status (yes or no)^2^, and flavor preference (yes or no)^3^ did not have an effect on the outcomes of the interest as a main effect or an interaction. Therefore, we conducted a two-way multivariate analyses of variance (MANOVA) to compare the group means of three vaping behaviors (days of vaping per week, number of vaping episodes per day, and number of puffs per vaping episode) by gender (males vs. females) and pandemic onset (pre- vs. during-pandemic). To establish pre- and during-pandemic behaviors, we asked respondents to report the three vaping behaviors prior to learning about the pandemic (pre-pandemic) and after learning about the pandemic (during-pandemic). For significant multivariate effects, we conducted univariate analyses to test the effect of pandemic onset, gender, and their interaction on each individual outcome. We then conducted paired *t-*tests for statistically significant univariate tests to test differences in vaping behaviors of each gender with respect to pandemic onset, and an independent samples *t*-test to test differences in vaping behaviors at each pandemic period (pre- vs. during-pandemic) between genders. A *p* < 0.05 indicated a significant effect. SPSS 26.0 was used for analysis.

## Results

### Sample Characteristics

Though 1,308 respondents completed the survey, only 1,209 (92.4%) reported using a nicotine-containing e-cigarette and only these participants were analyzed. 44.7% of respondents (*n* = 540) reported different vaping behaviors pre- vs. during-pandemic and 55.3% (*n* = 669) reported unchanged behaviors. Of those who reported different vaping behaviors after pandemic onset, (*n* = 540), 51.1% (*n* = 274) were female, 55.9% (*n* = 302) were youth aged 16–18, 56.7% (*n* = 306) were employed, and 88.6% (*n* = 453) preferred using flavored vape/e-juice.

Respondents who reported unchanged vaping behaviors after pandemic onset reported different vaping behaviors overall than respondents who reported changed behaviors pre- and during-pandemic, *F*_(3, 1, 205)_ = 24.20*, p* < 0.001, $\eta_{\text{p}}^{2}$ = 0.057. Compared to respondents who reported changed behaviors, those reporting unchanged behaviors reported a lower number of days of vaping per week [*M* = 6.51, *SD* = 1.38; *F*_(1, 1, 207)_ = 41.18, *p* < 0.001, $\eta_{\text{p}}^{2}$ = 0.033], number of vaping episodes per day [*M* = 5.93, *SD* = 1.86; *F*_(1, 1207)_ = 49.58, *p* < 0.001, $\eta_{\text{p}}^{2}$ = 0.039], but not number of puffs per vaping episode [*M* = 6.13, *SD* = 4.79; *F*_(1, 1207)_ = 0.001, *p* = 0.979]. Respondents reporting changed and unchanged behaviors were not different with respect to age [*t*_(1, 207)_ = 0.796, *p* = 0.426, 95% CI: −0.127–−0.30] or gender [*X*^2^ (1, *N* = 1,209), *p* = 0.079].

### Main Results

The within-subjects multivariate effect of pandemic onset was statistically significant, *F*_(3, 533)_ = 52.81, *p* < 0.001, $\eta_{\text{p}}^{2}$ = 0.229. Respondents reported a lower number of days of vaping per week, number of vaping episodes per day, and number of puffs per vaping episode. The between-subjects multivariate effect of gender on the three vaping outcomes was not statistically significant, *F*_(3, 533)_ = 2.14, *p* = 0.095, $\eta_{\text{p}}^{2}$ = 0.012. However, the multivariate interaction between pandemic onset and gender was significant, *F*_(3, 533)_ = 2.86, *p* = 0.036, $\eta_{\text{p}}^{2}$ = 0.016.

**Figure 1:**
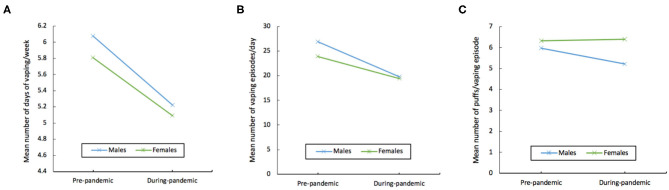
Interaction graphs illustrating mean values segmented by pandemic onset and gender of **(A)** number of days of vaping per week, **(B)** number of vaping episodes per day, and **(C)** number of puffs per vaping episode.

The effects at the univariate level are displayed in Table 1. There was a significant main effect of pandemic onset on all three vaping outcomes. With respect to the interaction of pandemic onset and gender, there was a significant main effect on number of vaping episodes per day and number of puffs per vaping episode, but not days of vaping per week (Figure 1A).

**Table 1:** Univariate effects for pandemic onset, gender, and their interaction for the three vaping outcomes.

		***F***	***p***	**$\eta_{\text{p}}^{2}$**
Pandemic onset	Days of vaping/week	117.61	0.000^*^	0.180
	Episodes of vaping/day	80.04	0.000^*^	0.130
	Puffs/vaping episode	3.91	0.048^*^	0.007
Gender	Days of vaping/week	1.37	0.242	0.003
	Episodes of vaping/day	0.52	0.47	0.001
	Puffs/vaping episode	3.43	0.065	0.006
Pandemic onset x Gender	Days of vaping/week	0.94	0.334	0.002
	Episodes of vaping/day	4.10	0.043^*^	0.008
	Puffs/vaping episode	5.73	0.017^*^	0.011

* *Indicates significant effect, p < 0.05*.

#### Vaping Behaviors pre- vs. During-Pandemic by Gender

A series of 2-tailed paired *t*-tests revealed that males and females responded differentially to pandemic onset (Table 2). Males reported fewer vaping episodes per day during-pandemic compared to pre-pandemic, *t*_(262)_ = 7.40, *p* < 0.001, 95% CI: 5.19–8.97 (Figure 1B). Males also reported fewer number of puffs per vaping episode during-pandemic than pre-pandemic, *t*_(263)_ = 3.23, *p* = 0.001, 95% CI:0.292–1.20 (Figure 1C). Females too reported fewer vaping episodes per day, albeit to a lesser extent than males, during-pandemic compared to pre-pandemic, *t*_(273)_ = 5.14, *p* < 0.001, 95% CI: 2.76–6.18 (Figure 1B). However, females did not reduce the number of puffs per vaping episode during-pandemic compared to pre-pandemic, *t*_(275)_ = −0.17, *p* = 0.868, 95% CI: −0.5.35−0.452 (Figure 1C).

**Table 2:** Mean vaping behaviors segmented by pandemic onset and gender.

	**Pre-pandemic (*****M, SD*****)**			**During-pandemic (*****M, SD*****)**		
	**Male**	**Female**	**Total**	**Male**	**Female**	**Total**
Days of vaping/week	6.08 (1.82)	5.81 (1.87)	5.94 (1.85)	5.22 (2.42)	5.09 (2.38)	5.15 (2.40)
Episodes of vaping/day	26.87 (29.46)	23.90 (27.39)	25.35 (28.43)	19.79 (26.84)	19.43 (27.09)	19.61 (26.94)
Puffs/vaping episode	5.96 (4.74)	6.32 (4.94)	6.14 (4.85)	5.21 (5.21)	6.39 (5.97)	5.81 (5.64)

#### Vaping Behaviors Pre-pandemic by Gender

Independent samples *t*-tests revealed that males and females were not different with respect to number of vaping episodes per day pre-pandemic, *t*_(538)_ = 1.24, *p* = 2.15, 95% CI: −1.76–7.82 (Figure 1B), or number of puffs per vaping episode pre-pandemic, *t*_(538)_ = −0.86, *p* = 0.39, 95% CI: −1.18−0.458 (Figure 1C).

#### Vaping Behaviors During-pandemic by Gender

The results of independent samples *t*-tests revealed that males and females were not different with respect to the number of vaping episodes per day during-pandemic, *t*_(535)_ = 0.11, *p* = 0.88, 95% CI: −4.21, 4.9 (Figure 1B). However, females reported more frequent puffs per vaping episode in comparison to males during-pandemic, *t*_(538)_ = −2.38, *p* = 0.017, 95% CI: −2.09-−0.200 (Figure 1C).^4^

## Discussion

The current study examined how youth and young adult e-cigarette users changed their vaping behaviors during the COVID-19 pandemic relative to the period preceding the pandemic. Further, the study findings shed light on gender differences in vaping behaviors in response to the pandemic. These findings are discussed in the context of the limited existing literature on this topic and the implications of the findings.

The main finding of the study is the reduced vaping behavior overall among participants who reported different vaping behaviors pre- and during-pandemic: lower days of vaping per week, lower episodes of vaping per day, and puffs per vaping episode. However, this finding should be considered alongside the fact that less than half of respondents indicated that they changed their vaping behaviors after learning about the pandemic. This is in line with the findings of another study that suggest that young (<21 years old) e-cigarette users in the United States who changed their vaping behaviors after pandemic onset were more likely to report decreased use than increased use, and also that roughly half of this sample reported different vaping behaviors after pandemic onset (26). There is both concern and promise in this finding. The concern is centered around the fact the findings depict small changes in vaping behaviors among youth and young adults during the COVID-19 pandemic, despite difficulties in accessing e-cigarettes (e.g., closed retail outlets, physical distancing, reduced social sourcing) (20–23). This suggests that efforts to reduce vaping, even in the midst of the pandemic, are crucial. Recent literature has identified potentially promising vaping cessation strategies for youth and young adults, such as traditional and mobile health counseling (29). Such strategies need to be urgently implemented. The promise in this finding, however, is related to visualizing the COVID-19 pandemic as an opportunity for effective health promotion messaging to youth and young adults that frames e-cigarette use as a preventable behavior that may increase risk of COVID-19 transmission and severity. Arguably, reduced vaping behaviors during the COVID-19 pandemic are at least partially attributed to the fear of potential complications from vaping if one were to contract the virus or simply the increased prospects of contracting the virus from vaping socially (e.g., sharing the devices). Recent research that demonstrated increased risk of COVID-19 among e-cigarette users reinforces this argument (10). Social marketing campaigns utilizing fear and self-efficacy messages may be an important approach to capitalize on in order to reduce vaping during the pandemic (30). The main finding of the study contradicts some findings on changes in substance use for other addictive products during the pandemic, namely the unchanged weekly consumption of alcohol, cannabis, and tobacco use among Canadians 15–34 years old (31). This difference may be explained by the increased cautionary messages from public health entities on the risks of vaping during the COVID-19 pandemic (24, 25). However, research conducted in other regions indicates that youth and young adults (>16 years old) in England increased alcohol use during the early months of the pandemic (32).

The second main finding is the differential response of females and males to the pandemic—in particular male, but not female, reductions in puffs per vaping episode during-pandemic. This is consistent with past research that demonstrated higher female receptivity to non-nicotinic elements of the vaping experience, including stress reduction (33). Further, stressors surrounding the pandemic, such as uncertainties in females' personal life [e.g., parenting; (34)], may keep female vaping consistent throughout the pandemic in comparison to pre-pandemic. The finding serves as an alert for the need of healthier coping mechanisms for females as recommended by prior research (33).

Two observations with respect to gender differences pre- and during-pandemic are worth noting. The first is the lack of differences between males and females in vaping behaviors pre-pandemic. This finding is consistent with the lack of gender differences in ever vaping (35). However, such studies tested differences in “ever use,” which is a prevalence measure, while we used measures of vaping frequency, which indicates how much youth and young adults use e-cigarettes (35). In this sense, our findings extend the literature by indicating that, besides lack of differences in ever use of e-cigarettes, vaping frequency is also not different among genders. Second, both males and females reported the same number of vaping episodes per day during-pandemic, despite females taking more puffs per vaping episode during-pandemic. This finding highlights an urgency for selective vaping cessation for females as they are more vulnerable to taking more puffs from their e-cigarette when they have the opportunity to use it during the pandemic relative to males. This is partially in line with the findings of at least one study that found that females consume more alcohol during the pandemic (36). Altogether females seem to be more vulnerable to higher substance use relative to males during the pandemic.

### Limitations

There are a number of limitations for the current study. First, we used a cross-sectional survey to examine vaping behaviors pre- and during-pandemic using retrospective measurement of past behaviors. This may lead to inaccuracies in self-reported behaviors by respondents. However, we conducted the survey within 2 months of the onset of the COVID-19 pandemic which minimizes recall bias and serves as a good first step toward understanding pandemic effects on vaping behaviors. Nevertheless, longitudinal studies may capture more precise changes in vaping behaviors. The cross-sectional nature of our study also limits what we can infer about which aspects of the pandemic encouraged respondents to change their vaping behaviors, especially participant characteristics that were not collected in our survey (e.g., whether participants are students). Second, our sample consisted of regular (at least once/week over the last 3 months) Canadian e-cigarette users aged 16–24, which may limit the generalizability of the results to users in other geographic regions, older users, and experimental e-cigarette users. However, we examined a sample of Canadians who are the most prevalent e-cigarette users (youth and young adults) from five diverse provinces. Further, vaping characteristics have been found to be reasonably universal across different regions (37). Third, though we assessed cigarette use and other substance use (alcohol and cannabis) among our sample, we did not examine changes in cigarette or other substance use pre- vs. during-pandemic. Thus, we cannot infer whether decreases in vaping behavior may have influenced cigarette or other substance use and vice versa. Fourth, our findings are limited to the early months of the pandemic and should not be extrapolated to the present and future days of the pandemic without further investigation.

## Conclusion

To conclude, the current study provides insight into how pandemic onset, alone and considered by gender, may have influenced vaping behaviors among Canadian youth and young adults. The still-concerning proportion of vaping behaviors among this demographic, even in the current pandemic, emphasize the need for immediate resources aimed at reducing or discontinuing use among youth and young adults, rather than prevention-focused efforts. As the pandemic continues to evolve, it is necessary to continue monitoring vaping behaviors among this population.

## Data Availability Statement

The datasets presented in this article are not readily available because ethics clearance was not obtained to provide a publicly available dataset. Requests to access the datasets should be directed to mohammed.al-hamdani@smu.ca.

## Ethics Statement

The studies involving human participants were reviewed and approved by Saint Mary's University Research Ethics Board. Written informed consent from the participants' legal guardian/next of kin was not required to participate in this study in accordance with the national legislation and the institutional requirements.

## Author Contributions

MA-H and DBH: Conceptualization, methodology, software, validation, formal analysis, investigation, writing—review and editing, and project administration. N/A: resources. DBH: Data curation, writing—original draft preparation, and visualization. MA-H: supervision and funding acquisition. All authors have read and agreed to the published version of the manuscript.

## Conflict of Interest

DBH and MA-H are employees of the Lung Association of Nova Scotia.

## Footnotes

^1^Main effect: *F*_(3,533)_ = 1.18, *p* = 0.318; Interaction: *F*_(3,533)_ = 1.45, *p* = 0.266.

^2^Main effect: *F*_(3,533)_ = 1.79, *p* = 0.147; Interaction: *F*_(3,533)_ = 1.04, *p* = 0.373.

^3^Main effect: *F*_(3,504)_ = 1.23, *p* = 0.300; Interaction: *F*_(3,504)_ =.106, *p* = 0.956.

^4^Equal variances not assumed, Levene's Test for Equality of Variances, *p* = 0.001.
